# Clay Nanomaterials Sorbents for Cleaner Water: A Sustainable Application for the Mining Industry

**DOI:** 10.3390/nano15151211

**Published:** 2025-08-07

**Authors:** María Molina-Fernández, Albert Santos Silva, Rodrigo Prado Feitosa, Edson C. Silva-Filho, Josy A. Osajima, Santiago Medina-Carrasco, María del Mar Orta Cuevas

**Affiliations:** 1 Department of Analytical Chemistry, Faculty of Pharmacy, University of Seville, E-41012 Seville, Spain; molinafernandezmaria@hotmail.com (M.M.-F.); asantos3@us.es (A.S.S.); rprado@us.es (R.P.F.); 2 Laboratório Interdisciplinar de Materiais Avançados, LIMAV, Programa de Pós-Graduação em Ciência e Engenharia de Materiais, PPGCM, Universidade Federal do Piauí, UFPI, Teresina 64049-550, Brazil; edsonfilho@ufpi.edu.br (E.C.S.-F.); josyosajima@ufpi.edu.br (J.A.O.); 3 X-Ray Laboratory (CITIUS), University of Seville, E-41012 Seville, Spain

**Keywords:** industrial water, decontamination, clay nanomaterials, sorption, ion exchange

## Abstract

The increasing shortage of drinking water, driven by reduced rainfall and the intensification of industrial and agricultural activities, has raised justified concerns about the quantity and quality of available water resources. These sectors not only demand high water consumption but also discharge large amounts of toxic substances such as organic matter, metal ions and inorganic anions, posing risks to both public health and the environment. This study evaluated the effectiveness of clay-based nanomaterials in the treatment of contaminated industrial wastewater from the mining sector. The materials tested included montmorillonite, high-loading expandable synthetic mica, and their organically functionalized forms (MMT, Mica-Na-4, C18-MMT, and C18-Mica-4). The experimental results show that these clays had minimal impact on the pH of the water, while a notable decrease in the chemical oxygen demand (COD) was observed. Ion chromatography indicated an increase in nitrogen and sulfur compounds with higher oxidation states. Inductively coupled plasma analysis revealed a significant reduction in the calcium concentration and an increase in the sodium concentration, likely due to cation exchange mechanisms. However, the removal of copper and iron was ineffective, possibly due to competitive interactions with other cations in the solution. Fourier transform infrared spectroscopy (FTIR) and X-ray diffraction (XRD) confirmed the structural modifications and interlayer spacing changes in the clay materials upon exposure to contaminated water. These findings demonstrate the potential of clay minerals as effective and low-cost materials for the remediation of industrial wastewater.

## 1. Introduction

Access to clean and safe water for human consumption is a growing global concern. Although the nature of water-related challenges varies across different regions, they generally stem from two main factors: physical scarcity, caused by limited rainfall or natural water sources, and excessive exploitation, particularly in industrial activities [1]. Rapid population growth and the intensification of industrial activities, such as the textile, pulp and paper, mining and petrochemical industries, have led to an exponential increase in the generation of complex effluents [2]. These effluents are frequently characterized by the presence of a wide range of pollutants, including toxic heavy metals, persistent organic dyes, pharmaceutical compounds, and refractory organic matter, which pose serious threats to aquatic ecosystems and human health [3]. In many cases, the discharge of these effluents occurs without adequate treatment, violating strict environmental regulations and exacerbating the global water crisis [4,5,6]. The urgency to find effective and economically viable solutions is amplified by increasing environmental awareness and stricter regulations on effluent discharge, posing continuous challenges to industries to adopt innovative sustainable treatment practices.

This scenario underscores the urgent need for efficient and affordable techniques capable of removing such contaminants and improving the water quality for reuse or safe discharge. A critical parameter for evaluating the effectiveness of effluent treatment and the extent of organic pollution is the chemical oxygen demand (COD). COD is widely used to assess the pollutant load of industrial effluents, and its reduction is a key objective in wastewater treatment [7]. Unlike biochemical oxygen demand (BOD), COD quantifies both biodegradable and non-biodegradable organic matter, providing a more comprehensive measure of the total pollutant load in industrial effluents [7]. COD measures the amount of oxygen required to chemically oxidize the organic and inorganic compounds present in water, serving as an essential indicator of the pollutant load and biodegradability of the effluent [7]. The presence of high COD levels in industrial effluents, such as those from the textile industry, indicates a high concentration of organic matter, which can deplete dissolved oxygen in water bodies, causing severe environmental impacts [7]. The complexity and recalcitrant nature of many organic compounds in industrial wastewater make COD removal a particularly difficult challenge, as many of these pollutants are resistant to conventional biological and chemical degradation [7]. Although conventional methods aim to reduce COD, challenges persist in meeting discharge standards. Achieving compliance with current discharge standards requires a comprehensive analysis of the effluent, the careful design of treatment systems, and proper control of operational conditions. However, conventional water treatment technologies are often costly and complex [8].

Various methods are currently employed to treat industrial and mining wastewater, each with specific advantages and limitations [9]. Technologies such as electrodialysis [10] and Fenton processes [11] offer high removal efficiency but involve high operational costs and technical demands. For example, the Fenton process, while effective in degrading refractory compounds and generating hydroxyl radicals for pollutant oxidation, is generally expensive due to the continuous need for reagents (such as hydrogen peroxide and iron salts) and the subsequent generation of large volumes of iron-rich sludge. This sludge, classified as hazardous waste, requires proper disposal, which significantly increases the operational and environmental costs of the process, limiting its large-scale application [7]. Other approaches, such as the use of aluminum-based coagulants, generate large volumes of sludge and may pose health risks with prolonged use. Furthermore, the inefficiency or high costs associated with these technologies limit their large-scale application, especially in developing countries [2]. Therefore, the development of new, low-cost, and environmentally friendly alternatives that are effective and sustainable has become a priority in scientific research [12,13]. The search for materials and treatment methods that minimize environmental impact and operational costs, while ensuring high efficiency in the removal of complex pollutants, is an active and globally relevant field of research. Sorption is a traditional mechanism that is particularly attractive for the treatment of wastewater with high organic loads. The sorption efficiency depends significantly on the sorbent material. Activated carbon is widely regarded as an effective adsorbent due to its high surface area and affinity for organic pollutants. Among the possible sources of activated carbon, those derived from agricultural waste present significant potential. Nonetheless, the high cost of activated carbon limits its use in large-scale industrial applications, driving the search for more affordable alternatives. To address these challenges, alternative sorbents need to be explored for their cost-effectiveness and high sorption potential [14,15,16].

In this context, nanomaterials based on natural clays have emerged as a promising and sustainable solution. Nanoclays are a broad class of natural or synthetic clay minerals with at least one dimension in the nano-range order and are characterized by a layered structure. Within the classification of nanoclays, those with cationic charge include montmorillonite and micas, as well as their organofunctionalized versions, which are the subject of this work [17,18]. Nanoclays are abundant and low-cost materials that exhibit excellent potential for use as unconventional sorbents in water treatment systems. Their effectiveness is attributed to properties such as their high specific surface area, porosity, ion exchange capacity, and structural versatility [19]. Among natural clays, montmorillonite (a type of smectite) and bentonite (a type of clay predominantly composed of montmorillonite) are widely studied for their 2:1 lamellar structure, characterized by an octahedral aluminum/magnesium layer sandwiched between two tetrahedral silicon layers [20,21]. This unique structure grants them a high specific surface area, varying, for example, around 28.17 m^2^/g for bentonite [20], and a remarkable swelling capacity, which allows for the expansion of the interlayer space and the intercalation of various molecules [20,22]. The versatility of montmorillonite and bentonite makes them ideal candidates for the removal of a wide range of pollutants in industrial wastewaters, including heavy metals, dyes, and organic matter, through mechanisms such as ion exchange (due to their high cation exchange capacity), surface sorption, and complex formation [3,20]. These characteristics, combined with their abundance and low cost, make natural clays an attractive alternative for effluent treatment, promoting sustainability and economy in water purification processes [2].

In addition to natural clays, synthetic clay minerals, such as high-charge micas (Na-Mica-x), have gained prominence due to their controlled and reproducible properties, which overcome some limitations of natural clays, such as the presence of impurities, variability in composition, and difficulty in standardization [18,22]. Synthetic micas (Na-Mica-x) are particularly notable for their high purity, high cation exchange capacity (CEC) of up to 468 cmol(+)/Kg, absence of secondary phases, and swelling capacity [22]. Their high interlaminar charge (up to 2, 3 or 4 per unit cell) results from isomorphic substitution at the tetrahedral site (substitution of Si^4+^ by Al^3+^), making them highly reactive and promising for selectively interacting with various organic and inorganic compounds [18,22,23]. The versatility of synthetic micas can be further enhanced by modifications with organic molecules, such as surfactants, silanes, and polymers, which alter their surface properties (e.g., hydrophobicity) and increase their sorption capacity for specific pollutants, making them effective in complex industrial effluent environments [22]. The engineering of these properties allows synthetic mica to be designed for specific applications, overcoming the inherent heterogeneity of natural clays and offering unprecedented control over the sorption process [22]. Clays, both natural and synthetic, are widely available, socially accepted, and require minimal maintenance, making them an attractive option for large-scale applications.

Over recent years, the application of clays in water purification systems has gained momentum, particularly due to their ability to remove contaminants through sorption, molecular sieving, and ion exchange. These mechanisms allow for the reduction of organic matter, toxic compounds, and heavy metals in polluted waters [22,24,25,26,27]. However, despite the general interest in clay-based sorbents for various pollutants, studies specifically investigating their direct efficacy in comprehensive COD removal from complex industrial wastewaters, and the detailed structural modifications of the clays after this process, remain limited. Most studies have focused on the removal of specific dyes or heavy metals, often in synthetic solutions, without an in-depth evaluation of the overall impact on the COD of real effluents and the structural transformations of the clays resulting from this sorption [2,3].

This is particularly true for high-charge synthetic micas, where research to date has demonstrated their versatility in adsorbing various contaminants, such as metals (e.g., Hg^2+^, Pb^2+^, Cd^2+^), pharmaceuticals (e.g., ibuprofen), perchlorate, and non-ionic hydrocarbons (e.g., toluene, benzene, phenol) [22,23]. However, a comprehensive understanding of their performance in COD removal from complex industrial effluents and the associated structural modifications is still developing [22]. For montmorillonite and bentonite, although they have demonstrated potential in COD removal in various studies, often via adsorptive mechanisms or combined with advanced oxidation processes [3,20], the focus has not always been on the intrinsic adsorptive capacity for a broad spectrum of organic compounds contributing to COD, nor on the detailed structural evolution of the clay minerals themselves during this process in real industrial environments [2,20]. A detailed analysis of the structural and morphological modifications of clays after exposure to complex effluents is crucial for understanding the removal mechanisms and the long-term stability of sorbents, but this area remains underexplored in relation to COD removal [20]. This research gap is significant because the behavior of sorbents in real systems can be substantially different from that observed under controlled laboratory conditions, influenced by the presence of multiple contaminants and the complexity of the effluent matrix [2]. Furthermore, the reusability of sorbents is a crucial economic and environmental factor that directly depends on understanding post-sorption structural changes; without this knowledge, the optimization of regeneration processes and long-term viability are limited [3]. The elucidation of these mechanisms and the correlation between the clay structure and its COD removal capacity in complex effluents represent a significant advancement in the field of wastewater treatment, offering guidelines for the development of more efficient and sustainable sorbents [22].

The objective of this study was to investigate the use of mineral clays for the treatment of industrial wastewater, focusing on both the changes in water composition (including a comprehensive analysis of COD reduction) and the structural modifications of the clays before and after exposure. This study aims to address the aforementioned research gap by providing a deeper understanding of the mechanisms of COD removal by mineral clays, specifically natural variants (montmorillonite and bentonite) and synthetic ones (high-charge micas), and evaluating their potential as a sustainable and cost-effective solution for complex industrial wastewater treatment, highlighting the novelty of this approach.

## 2. Experimental Section

### 2.1. Materials and Reactives

The raw water used in this research was wastewater from a mining industry located in the south of Spain.

The montmorillonite (MMT) used came from Patagonia (Rio Negro, Argentina) and was supplied by Catiglioni Pes and Co (Buenos Aires, Argentina). The chemical composition was [(Si_3.83_Al_0.11_) (Al_1.43_ Fe^3+^_0.26_Mg_0.30_) O_10_ (OH)_2_]Na_0.30_Ca_0.09_K_0.01_, the mineral composition was Na-montmorillonite (>99%) with quartz and feldspar as minor phases [28], and the cation exchange capacity (CEC) was 0.8250 mmol/g clay [29].

The precursors for the synthesis of high-charge synthetic mica (Mica-Na-4) were SiO_2_ (Sigma Aldrich, St. Louis, MO, USA), CAS No. 112945-52-5, purity 99.8%), Al(OH)_3_ (Sigma Aldrich; CAS No. 21645-51-2), MgF_2_ (Sigma Aldrich; CAS No. 7783-40-6), and NaCl (Sigma Aldrich; CAS No. 7647-14-5, purity ≥ 99.5%).

### 2.2. Synthesis of Mica-Na-4

**Figure 1:**
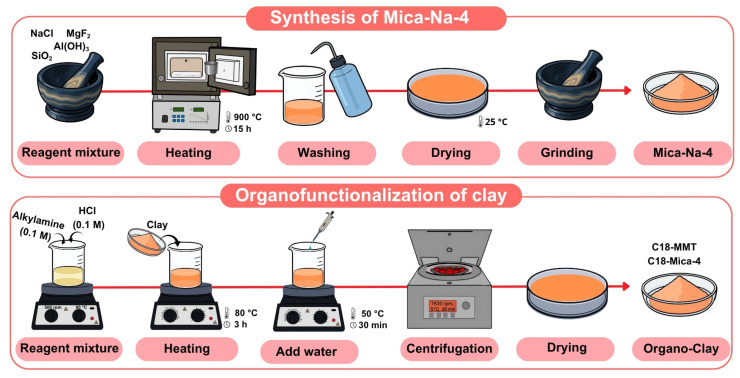
Diagram of the synthesis of Na-mica-4 and the organofunctionalization process of the clays.

**Figure 2:**
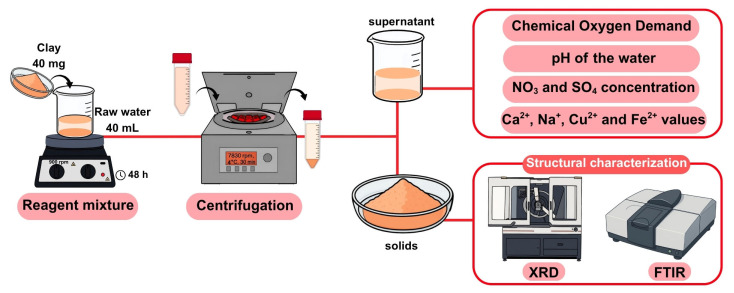
Diagram of the experimental setup.

For the synthesis of Mica-Na-4, we used the procedure described by Alba et al., 2006 [30]. For this, the reagents SiO_2_, Al(OH)_3_, MgF_2_ and NaCl were weighed and mixed in an agate mortar. The mixture was then introduced into a platinum crucible and heated in a muffle at 900 °C for 15 h; the heating ramp used was 10° min^−1^. Subsequently, it was washed with abundant deionized water, dried at room temperature and ground again in an agate mortar (Figure 1). The synthesized Mica-Na has a CEC of 4.68 mmol g^−1^ and its structural formula is Na_4_[Si_4_Al_4_]Mg_6_O_20_F_4_]·nH_2_O. Finally, its correct synthesis was verified via characterization with X-ray diffraction (see Section 2.7 and Figure 2).

### 2.3. Organofunctionalization of MMT and Mica-Na-4

Organoclays were synthesized via cation exchange reactions [31] between Mica-Na-4 or MMT with an excess of alkylamine of 18 carbon atoms (Sigma Aldrich, CAS no. 124-30-1, purity ≥ 99.0%), 2.5 times the CEC of the clay [32].

Alkylamine was added to an equivalent amount of HCl (0.1 M) for its protonation; the mixture was stirred at 500 rpm for 3 h at 80 °C. Subsequently, Mica-Na-4 or MMT was added and maintained for 3 h with stirring at 80 °C. Then deionized water was added at 50 °C and stirred for 30 min, and the dispersion was centrifuged at 7830 rpm for 30 min at 5 °C. Finally, the solids were allowed to dry at room temperature (Figure 1) and were characterized using X-ray diffraction (Figure 2). The synthesized organoclays were identified as C18-MMT and C18-Mica-4.

### 2.4. Sample Collection

The raw water samples were taken at the discharge point of the industrial water treatment plant of a mining company located in the south of Spain; all were collected in 1 L plastic cans that had previously been labelled and rinsed. They were kept refrigerated until they were in contact with the clays under study in less than 24 h.

### 2.5. Sorption Process

In total, 40 mg of each of the four clays was weighed and placed in contact with 40 mL of raw water in a glass bottle for 48 h in a shaker at 900 rpm. A fixed clay-to-water ratio of 1:1 (40 mg of clay to 40 mL of raw water) was selected based on its frequent use in the literature for evaluating the performance of clay materials in batch adsorption systems [33,34,35]. Ratios of 1:1 and 2:1 are commonly employed as standard conditions in initial screening experiments with clays such as montmorillonite and synthetic micas. Varying the clay-to-water ratio can significantly influence adsorption efficiency. A higher clay dosage typically increases the number of available active sites, potentially improving removal efficiency, especially when target contaminants are present at relatively high concentrations. However, beyond a certain threshold, the benefit may diminish due to particle agglomeration, reduced mass transfer, or the saturation of active sites. Conversely, lower clay dosages may lead to an insufficient active surface area, resulting in reduced removal efficiency [36,37]. After the contact time, the suspensions were centrifuged at 7830 rpm and 4 °C for 30 min. The supernatant and solids were separated for subsequent analysis (see Section 2.6 and Figure 2) and structural characterization (see Section 2.7 and Figure 2), respectively.

Finally, the clay that provided the best results, Mica-Na-4 (see Section 3.1), was selected for further testing. Following the procedure described above, raw water was brought into contact with Mica-Na-4 for 1, 10, 20 and 30 min, in order to evaluate the potential sorption processes at these contact times. All experiments were conducted in duplicate and under standard temperature and pressure conditions.

### 2.6. Analysis of Water

See Figure 2.

pH was measured using a pH meter (Crison, Alella, Spain).

To measure the chemical oxygen demand (COD), the HI94754 reagent kit and HANNA photometer were used (Hanna, Leighton Buzzard, UK), covering a range from 0 to 1500 ppm of COD O_2_.

The determination of sulphates and nitrates was carried out in the Microanalysis Service located in the Research, Technology and Innovation Centre of the University of Seville (CITIUS Celestino Mutis), and according to the internal procedure, the determinations were carried out by ion chromatography. The equipment used was a Metro brand ion chromatograph, model 930 Compact IC (Herisau, Switzerland), with chemical and sequential suppression; a Metrosept A Supp 7 column, Metrohm (Herisau, Switzerland), 3.6 mM Na_2_CO_3_, was used as eluent at a flow of 0.8 mL/min and at 45 °C. The chromatography was carried out in 35 min, where the retention times of the analytes were 19.18 min for nitrates and 30.32 min for sulfates.

The determination of Ca, Cu, Fe and Na was also carried out in the Microanalysis Service (CITIUS Celestino Mutis). The sample was diluted prior to ICP analysis following an internal procedure. For this purpose, an atomic emission spectrometer with an inductively coupled plasma excitation source and a CCD optical detection (ICP-OES-SPB-TI), Spectroblue TI model (Ametek Inc., Kleve, Germany) was used. A wavelength range between 165–770 nm, a cross-flow nebulizer, axial plasma viewing mode, a plasma power of 1350 W, a coolant flow of 14 L min^−1^, an auxiliary flow of 1.20 L min^−1^, and a nebulizer flow of 0.82 L min^−1^ were used. Three measurement replicates were performed, and the wavelengths of the measured elements were 315.887 nm for calcium, 327.396 nm for copper, 259.941 nm for iron, and 588.995 nm for sodium.

### 2.7. Solid Characterization

See Figure 2. The clays before and after contact with water were analyzed in the X-Ray Laboratory located in the Research, Technology and Innovation Centre of the University of Seville (CITIUS I) in a Bruker D8 Advance A25 diffractometer (Bruker, Karlsruhe, Germany) in Bragg–Brentano configuration, using a Lynxeye PSD detector (Bruker, Karlsruhe, Germany) equipped with a Kα copper radiation source (wavelength 0.15405 nm). The diffractograms were acquired between 1° and 70° 2θ, step of 0.03°, time per step of 0.1 s, tube conditions of 40 kV and 30 mA, and in the Microanalysis Service (CITIUS Celestino Mutis) where, using an internal procedure, they performed infrared analyzes with Fourier transform. The equipment used was a Bruker brand spectrometer, Invenio X model, with diamond ATR (Bruker Optics, Billerica, MA, USA). The parameters of the method were: resolution: 4 cm^−1^, number of scans: 32, scan from 4000 to 400 cm^−1^, type of source: MIR, RT-DLa TGS detector, beam splitter: KBr and scan speed: 20 Khz.

## 3. Results and Discussion

### 3.1. Influence of Clays on the COD Values of the Water Samples Tested

**Figure 3:**
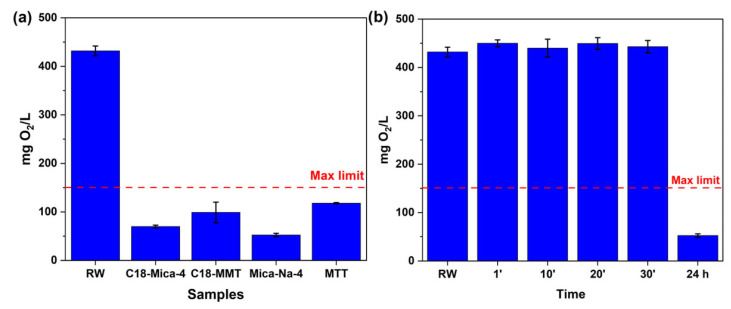
(**a**) COD values of industrial water before (RW) and after 24 h of contact with the four clays tested and (**b**) after contact with Mica-Na-4 for 1, 10, 20, and 30 min and 24 h.

In the present study, four different types of clays were evaluated for their effectiveness in reducing the chemical oxygen demand (COD) after different contact times with water. After 24 h of contact, all the clays managed to reduce COD to values below 150 mg O_2_ L^−1^, the maximum limit allowed by the Discharge Regulation for the Public Hydraulic Domain of Andalusia (Spain) [38]. These results indicate that the clays studied are viable for meeting regulatory requirements and minimizing the environmental impacts of liquid effluents (Figure 3a).

COD is widely recognized as a fundamental parameter for assessing the pollutant load in wastewater, providing representative information about the presence of oxidizable organic and inorganic compounds, thus making it essential for monitoring wastewater treatment processes [14,39]. Reducing COD is a key objective in effluent management, as high levels can severely compromise water quality in water bodies and threaten aquatic ecosystems [14]. Conventional methods, such as the Fenton process, while effective in degrading refractory compounds, are generally expensive due to the continuous need for reagents and the subsequent generation of large volumes of iron-rich sludge, which is classified as hazardous waste [7]. In contrast, sorption onto clays is considered an efficient and sustainable technique due to their high sorption capacity for organic matter, accessibility, and low cost, contributing significantly to the reduction of COD in wastewater [27,34,40].

Among the clays tested, Mica-Na-4 demonstrated the highest effectiveness in reducing COD after 24 h of contact. This superior performance may be related to its specific characteristics, such as its larger surface area and the presence of functional groups that favor interactions with organic compounds [20]. As highlighted by Pazos et al., (2017) [41], the organo-functionalization of high-charge synthetic micas provides effective sorption capacity for non-ionic organic pollutants at the interface, and their sorption performance is a function of alkylammonium properties, such as chain length and the organization of the organic cation in the interlayer space. The high cation exchange capacity (CEC) of Mica-Na-4, which is 4.68 mmol g^−1^, is significantly higher than that of clays like montmorillonite (0.8250 mmol g^−1^), allowing for the greater availability of exchange sites to interact with the organic and inorganic load present in the effluent, justifying its superiority in COD removal [22]. The results presented in Figure 3b show that, at contact times of up to 30 min, reductions in COD are still low, indicating that the system has not yet reached sorption equilibrium during this initial period. These data reinforce the idea that equilibrium time for maximum efficiency occurs over longer periods, with 24 h of contact necessary for the clays to reach their maximum capacity for removing organic matter from water, as evidenced by the significant reduction in COD at this point (Figure 3b). The need for a prolonged contact time is common in sorption processes involving complex effluents, where the diffusion of analytes to the clay’s sorption sites can be slow [20]. In a similar study, Martín et al. (2018) [34] observed that, for pharmaceutical compounds and some industrial pollutants, a period of 24 h was necessary to achieve sorption equilibrium on organofunctionalized micas (C18-Mica-4), with compounds retained in the mica for at least seven days after extraction, which corroborates the sorption kinetics observed in this research.

The selection of contact times up to 30 min, followed by a long-term measurement at 24 h, was intended to evaluate both the initial adsorption kinetics and the potential equilibrium state of the system. This approach is commonly used in adsorption studies to distinguish between rapid surface adsorption processes and slower mechanisms influenced by pore diffusion or intra-particle transport [36,42]. While a significant reduction in COD was observed over the treatment duration, no measurements were taken between 30 min and 24 h, preventing the precise determination of the time at which the COD concentration falls below the regulatory limit of 150 mg O_2_/L. This represents a limitation of the present study. Future work will focus on narrowing this time interval to better define the minimum effective contact time required for practical applications.

Moreover, it is important to consider that COD reduction via sorption onto clays is subject to various experimental conditions, including the initial concentration of organic matter and pH [14].

### 3.2. Influence of Clays on the pH Values of the Tested Waters

In all samples, both before and after contact of the water with clay (Table 1) and at different times of contact with Mica-Na-4 (Table 2), the pH was measured; there were no significant variations in the pH values in any sample, thus confirming that contact with clay does not influence the pH of the water. In general, acidic pH favors sorption of inorganic compounds in clays [43]. Maintaining pH stability during the process is an important characteristic, as it ensures that the use of clays does not undesirably alter the chemical quality of the water. This is crucial not only to avoid impacts on subsequent treatment processes but also to protect the receiving environment, ensuring that the treated effluent maintains an acceptable pH range for discharge or reuse.

### 3.3. Influence of Clays on the Nitrate and Sulphate Values of the Tested Waters

**Figure 4:**
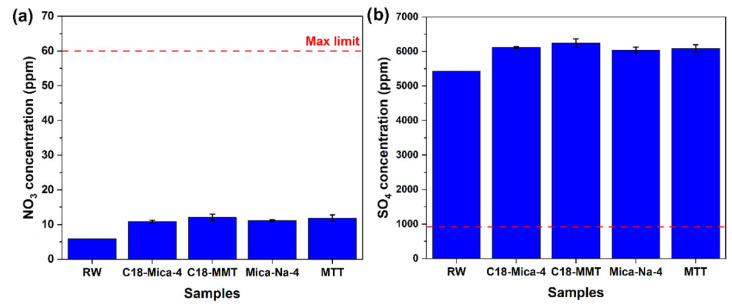
(**a**) Nitrate and (**b**) sulphate concentration values of industrial water before (RW) and after 24 h of contact with the four clays tested.

The clays tested do not decrease the concentration of nitrates but rather increase it (Figure 4a). This increase could be explained by the oxidative effects of the nitrites present in the water.

In previous studies, other researchers found that unmodified montmorillonite was ineffective in reducing nitrates [44], and that, if modified, the reduction efficiency was increased [45]. In this work, the efficacy of organofunctionalized MMT was analyzed. Figure 4a shows that the nitrate concentration increases slightly more than with natural MMT.

On the other hand, we also studied the behavior of Mica-Na-4 and, in turn, this organofunctionalized; since no previous studies related to these clays and this parameter have been found, no difference is observed between them. Although the presence of these clays increases the concentration of nitrates, it is still below the maximum limit allowed, so this would not be a limiting factor when using these clays for the treatment of this water.

After the clays came into contact with industrial water, an increase in sulphate concentration was also observed, being in all cases above the maximum allowed level of 900 ppm (Figure 4b). The increase in sulfate concentration after contact with these clays is a phenomenon that has also been observed in other studies. For example, Bentonite, a clay composed mainly of montmorillonite, demonstrated an increase in sulfate concentrations in water [46]. This behavior can be attributed to oxidation processes. Given that the treated water contains a high number of thiols, the increase in sulfates could be explained by the oxidative effect of the clays on these sulfur compounds, transforming them into sulfates. However, it is important to note that, although the increase in sulfates may be a result of the clay’s interaction with the effluent matrix, the final concentration exceeded the permissible limit, indicating the need for complementary treatment for sulfate removal or the exploration of clays with sorption or ion exchange mechanisms that are selective for sulfate removal.

### 3.4. Influence of Clays on the Values of Ca^2+^, Na^+^, Cu^2+^ and Fe^2+^ in the Tested Waters

**Figure 5:**
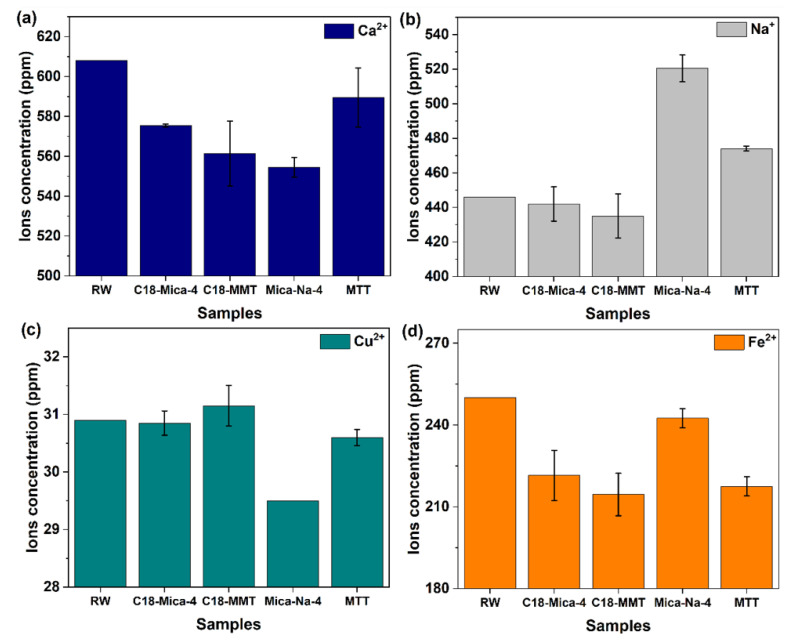
(**a**) Ca^2+^, (**b**) Na^+^, (**c**) Cu^2+^ and (**d**) Fe^2+^ concentration values of industrial water before (RW) and after 24 h of contact with the four clays tested.

The excess negative charge in the interlamellar space of both MMT and Mica-Na-4 is efficiently compensated by sodium ions, which occupy these spaces to maintain electrostatic neutrality [22]. In the case of Mica-Na-4, the greatest reduction observed in the calcium concentration in the industrial water after contact with the clay (Figure 5) can be explained by a cation exchange mechanism, where calcium ions present in the aqueous solution are replaced by sodium ions from Mica-Na-4. This process occurs due to the higher cation exchange capacity (CEC) of Mica-Na-4, which is 4.68 mmol/g, compared to other clays. The higher CEC of Mica-Na-4 allows for a greater capacity to adsorb cations, such as calcium, thereby promoting a greater reduction in its concentration in the water [47].

Additionally, the cation exchange between calcium and sodium in Mica-Na-4 explains the significant increase in sodium concentrations observed in the water. This increase is a direct consequence of the replacement of calcium cations by sodium ions, which results in the release of sodium into the solution. This phenomenon highlights the importance of CEC in modulating sorption and ion exchange processes, demonstrating the potential of Mica-Na-4 in the selective removal of calcium ions, while increasing the sodium concentration in the aqueous solution.

In the presence of C18-MMT and C18-Mica, a reduction in the calcium concentration present in water is also observed. Although there are not many studies that detail the exact mechanism of calcium sorption by organoclays modified with alkylammonium compounds, a possible interpretation for the reduced calcium in these materials could be the cation exchange of residual sodium traces that were not completely exchanged with primary amines during the organo-functionalization process [41]. The results of XRD in Section 3.5, show the presence of a new phase attributable to brushite (CaPO_3_(OH)·2H_2_O), which could contribute to the decrease in Ca in the water after contact with clay.

The clays tested did not allow the copper concentration values to fall below the allowed limits. In the bibliography, there are data from other clays and even modified montmorillonite that manage to reduce the concentration of copper ions by up to 80% [48]. Studies demonstrate the competitive effect on copper sorption in MMT in the presence of other cations such as Fe and Ca [49,50]. Although the value is above the acceptable level, Mica-Na-4 was the one that contributed the most to the reduction in Cu, which may have its removal inhibited by the presence of a multiphase pollutant medium [51].

The clays used in this test are capable of reducing iron concentrations; although they did not reduce it a sufficiently large amount to obtain a final concentration below the maximum allowed discharge limit, this response was expected, since, as has been shown in other studies, the presence of metal cations decreases iron sorption by clays. Cations such as zinc, lead, nickel, chromium, copper, etc., are predominant in the type of water studied [52]. The inefficiency in the complete removal of copper and iron can be attributed to the complex nature of industrial mining effluent, which contains a mixture of multiple metal ions and organic compounds. In such environments, competition for sorption sites on clays becomes a limiting factor [49]. For example, studies with sorbents for Cr(VI) removal in industrial effluents [53] showed that the presence of other metal ions such as Ni^2+^, Cu^2+^, Co^2+^ and Zn^2+^ can influence sorption, although the Ppy-OMMT NC3 nanocomposite demonstrated selectivity for Cr(VI). Furthermore, the presence of sulfates (Figure 4b), which is high in the tested water, may have an inhibitory effect on the sorption of metal cations due to complex formation or competition for sorption sites. Houmia et al. (2025) [54] also observed that, in mixtures of natural clays for heavy metal removal, the removal efficiency of Cu(II) was slightly lower compared to Zn(II) and Co(II) in environments with common competing ions, suggesting that copper may be more challenging to remove in multiphase systems.

### 3.5. Structural Changes in Clays

**Figure 6:**
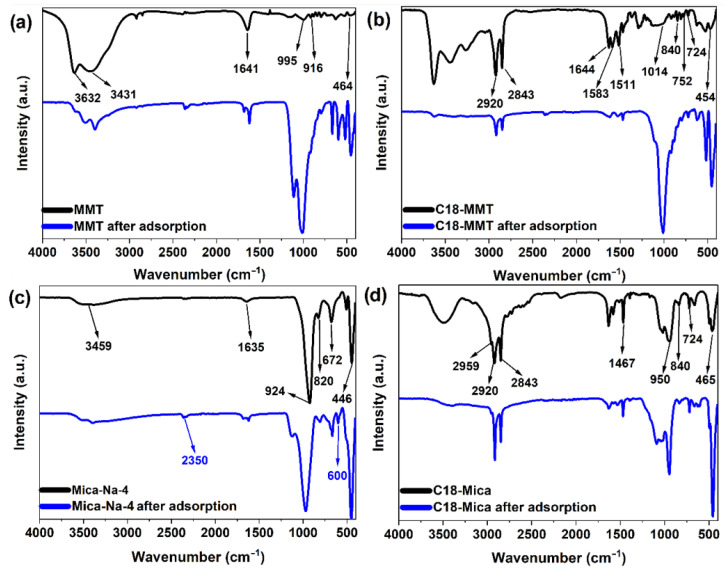
FTIR spectrum before (black) and after (blue) contact of RW (**a**) MMT, (**b**) C18-MMT, (**c**) Mica-Na-4, and (**d**) C18-Mica.

The FTIR spectrum of MMT (Figure 6a) shows characteristic peaks of the hydroxyl groups, silicates and cations present in the structure of montmorillonite [55]. The OH stretch region is dominated by a band centered at 3632 cm^−1^, accompanied by a wider band at 3431 cm^−1^ assigned to water stretch vibrations, and a narrower band at 1641 cm^−1^ assigned to water deformation [56]. The out-of-plane Si-O stretch band appears at 983 cm^−1^ and another small band is observed at 995 cm^−1^; it has been attributed to the Si-O stretch modes in the plane. The peaks between the wavelengths of 950–450 cm^−1^ correspond to the structural groups δAlOHAl, δAlOHFe, δAlOHMg, vSiO, δAlOSi, and δSiOSi. The signal at 800 cm^−1^ has been assigned to the Si-O vibration for quartz impurities [55]. After contact between the water and the clay, a peak appears at 2350 cm^−1^ that could correspond to the presence of traces of CO_2_ [57].

In the FTIR spectra of C18-MMT (Figure 6b), in addition to the bands corresponding to the MMT seen in the figure above, the bands corresponding to the stretch vibrations of the C-H bonds of the surfactants are observed at ~2920 and 2843 cm^−1^, corresponding to the asymmetric and symmetric stretching, respectively, of the -CH_2_ groups [29]. Confirming the intercalation of the surfactant in the C18-MMT, in the region from 1750 to 1350 cm^−1^, the appearance of the bands corresponding to the flexion modes of the alkyl groups (δ_as_CH_3_N and δ_as_CH_2_ at approximately 1583 and 1511 cm^−1^, respectively) is observed. The band at 1644 cm^−1^ corresponds to the deformation of the water molecule but appears with less intensity due to the displacement caused by the primary amine [58]. C18-MMT presents structural changes after contact with water, as the peak reappears at 2350 cm^−1^ as in MMT after sorption and, in addition, peaks located at 1200–1350 cm^−1^ disappear, which have not been identified in the literature. The area between 1400 and 600 cm^−1^ is complex, because there is a combination of elongation as well as bending vibrations.

The Mica-Na-4 has absorption bands related to tension and flexural vibrations of the OH group of the water located at 3459 and 1635 cm^−1^, respectively (Figure 6c). In addition, bands at 924 and 672 cm^−1^ are observed that correspond to the asymmetric and symmetrical tension vibration modes of the Si-O-Si link and another band at 446 cm^−1^ that has been attributed to the bending vibration of the Si-O-Si network. The peak at 820 cm^−1^ corresponds to the bending vibrations of the Al-Si-O bond [59,60]. After the sorption test, a signal reappears at 2350 cm^−1^ as in the previous clays, and a peak at 600 cm^−1^ is also observed.

C18-Mica (Figure 6d), in addition to having the same bands as Mica-Na, has two bands at 2920 and 2843 cm^−1^ due to v_as_(CH_2_) and v_s_(CH_2_) and one band at 2959 cm^−1^ due to v_as_(CH_3_) [32,41]. The band at approximately 1469 cm^−1^ is assigned to δ(CH_2_). In this case, in the FTIR spectrum of C18-Mica after sorption, the peak reappears at 600 cm^−1^ as observed for Mica-Na-4, although it does not seem to show any peak above 2350 cm^−1^, as was the case in the other clays tested.

**Figure 7:**
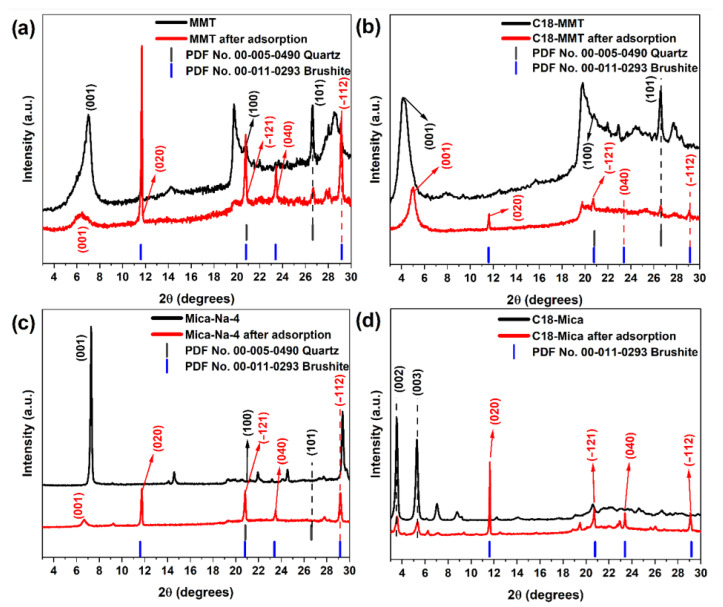
XRD of (**a**) MMT, (**b**) C18-MMT, (**c**) Mica-Na-4 and (**d**) C18-Mica before and after contact of RW.

The XRD pattern of raw montmorillonite (MMT) (Figure 7a) shows a prominent reflection at low angle (~6.7° 2θ), corresponding to the basal plane (001), with an interlayer spacing (d001) of approximately 1.253 nm [61,62]. After the sorption process, this peak shifts to a lower angle (~6.4° 2θ), indicating an increase in interlayer spacing to 1.382 nm. This expansion suggests the intercalation of species, possibly water molecules or adsorbed pollutants, between the clay layers. Such interlayer swelling may be associated with the hydration of exchanged cations and/or the presence of complexed species, which aligns with the observed decrease in Ca, Fe, and Cu content and the increase in Na. Furthermore, new diffraction peaks appear that match the phase of b(CaPO_3_(OH)·2H_2_O, PDF 00-011-0293), reinforcing the hypothesis of precipitation or complexation involving calcium during the sorption process. The presence of quartz (SiO_2_, PDF 00-005-0490) was also detected at a low intensity, as typically found in natural clays [28].

In the organically modified sample C18-MMT (Figure 7b), the initial interlayer spacing is 2.125 nm, indicating successful intercalation of alkylamine chains. After sorption, this spacing decreases to 1.778 nm, suggesting a structural reorganization of the intercalated organic chains. This partial collapse may be attributed to a reduction in the tilt angle of the alkyl chains caused by solvation and rearrangement upon contact with aqueous solution, as reported in previous studies [30,34]. The formation of brushite after sorption was again observed, indicating a similar interaction pattern as with unmodified MMT. Martín et al., (2018) [34] also observed a decrease in interlayer spacing and the degree of the tilt angle between the alkylammonium axis and the solid surface in C18-Mica-4 after the sorption of emerging pollutants, which suggests the reorganization of alkylammonium chains in the interlayer space of the organomica to accommodate adsorbed molecules.

For the Mica-Na-4 sample (Figure 7c), the (001) reflection shifts from 7.3° to 6.6° 2θ, corresponding to an increase in the interlayer spacing from 1.214 to 1.337 nm after sorption. In addition to this structural expansion, the XRD patterns show the emergence of brushite and quartz phases, again reflecting the retention of phosphate compounds and possible cation exchange during the sorption process. The formation of brushite (CaPO_3_(OH)·2H_2_O) is an important mechanism for the removal of calcium and potentially phosphates from industrial water [63,64]. This secondary phase, detected in XRD analyses, indicates that calcium ions are not only exchanged but can also be incorporated into new mineral structures on the clay surface, contributing to removal efficiency. Houmia et al. (2025) [54] also confirmed brushite formation in natural clay mixtures after heavy metal sorption, reinforcing the relevance of this mechanism.

Finally, the C18-Mica sample (Figure 7d) also displays structural changes after contact with the aqueous solution. Reflections corresponding to the (002) and (003) planes suggest a slight reduction in basal spacing, indicating the partial reorganization of the intercalated organic chains [27,65]. The detection of brushite in this sample confirms the trend observed across all systems, indicating strong interactions between the sorbents and calcium and phosphate ions.

These results indicate that, in addition to ion exchange and intercalation mechanisms, the formation of secondary phases such as brushite plays an important role in pollutant retention [66,67]. The observed variations in interlayer spacing highlight the role of surface functionalization in modulating both the structural and adsorptive behavior of the clay-based materials.

## 4. Conclusions

The present study demonstrates that clay mineral nanomaterials are an innovative, cost-effective, and sustainable option for the pre-treatment of complex industrial effluents. After 24 h of contact, every clay tested reduced the chemical oxygen demand (COD) to below 150 mg O_2_ L^−1^, thereby meeting the discharge limit set by Andalusia’s Public Hydraulic Domain Regulation. This performance is driven chiefly by Mica-Na-4, whose cation-exchange capacity (4.68 mmol g^−1^) is roughly 5.7 fold higher than that of montmorillonite (0.825 mmol g^−1^), enabling greater sorption of the organic and inorganic load. Throughout the treatment, the pH remained virtually unchanged (2.53 in the raw effluent versus 2.40–2.58 after treatment), confirming that the clays do not disrupt downstream pH-sensitive operations. Although nitrate concentrations rose slightly, they stayed within legal limits, whereas sulfate concentrations exceeded the 900 ppm threshold—evidence that a complementary sulfate-removal step is required. Ion-exchange data revealed a selective Na^+^ ↔ Ca^2+^ mechanism: calcium concentrations fell markedly while sodium increased, yet Cu^2+^ and Fe^2+^ removal was modest, a shortfall attributed to competition from other multivalent cations. X-ray diffraction showed basal-spacing expansions (e.g., MMT 1.253 to 1.382 nm; Mica-Na-4 1.214 to 1.337 nm) and the formation of brushite (CaPO_3_(OH)·2H_2_O), supporting the joint roles of ion exchange and precipitation in contaminant retention. Organically modified clays (C18-MMT, C18-Mica) experienced partial interlayer collapse (2.125 to 1.778 nm in C18-MMT) as alkylammonium chains reorganized around sorbed molecules, enhancing affinity for non-ionic organics. Kinetic data indicated that 30 min is insufficient to reach sorption equilibrium, whereas 24 h ensures maximum COD removal in such complex matrices. Taken together, these findings position Mica-Na-4 as the most promising sorbent for simultaneous organic-load reduction and Ca^2+^ removal, while highlighting the need for hybrid schemes, such as post-treatment sulfate polishing or targeted metal-ion sorbents, to meet all regulatory parameters. Future work should optimize continuous-flow contact times, explore regeneration and reuse of the clays, and integrate selective sulfate or heavy-metal removal to achieve fully compliant, circular treatment trains.

## Figures and Tables

**Table 1:** pH values before and after water contact with different clay minerals.

	RW Raw	C18-Mica-4	C18-MMT	Mica-Na-4	MMT
pH	2.53	2.48	2.55	2.43	2.58

**Table 2:** pH values before and after water at different contact times with Mica-Na-4.

	RW Raw	1 min	10 min	20 min	30 min	24 h
pH	2.46	2.40	2.52	2.47	2.51	2.43

## Data Availability

Data is contained within the article.
